# Dynamics of nano-confined water in Portland cement - comparison with synthetic C-S-H gel and other silicate materials

**DOI:** 10.1038/s41598-017-08645-z

**Published:** 2017-08-15

**Authors:** Guido Goracci, Manuel Monasterio, Helen Jansson, Silvina Cerveny

**Affiliations:** 1 Centro de Física de Materiales (CSIC, UPV/EHU)-Materials Physics Center (MPC), Paseo Manuel de Lardizábal 5, 20018 San Sebastián, Spain; 2 Shenzhen Advanced Civil Engineering Technology, Association Research Center Shenzhen Institute of Information Technology, Shenzhen, 518172 China; 3 State Key Lab of Advanced Welding Production Technology, Harbin Institute of Technology Shenzhen Graduate School, Shenzhen, 518055 China; 4 Department of Civil and Environmental Engineering, Chalmers University of Technology, Göteborg, Sweden; 5 Donostia International Physics Center, DIPC, 20018 San Sebastián, Spain

## Abstract

The dynamics of water confined in cement materials is still a matter of debate in spite of the fact that water has a major influence on properties such as durability and performance. In this study, we have investigated the dynamics of water confined in Portland cement (OPC) at different curing ages (3 weeks and 4 years after preparation) and at three water-to-cement ratios (*w*/*c*, 0.3, 0.4 and 0.5). Using broadband dielectric spectroscopy, we distinguish four different dynamics due to water molecules confined in the pores of different sizes of cements. Here we show how water dynamics is modified by the evolution in the microstructure (maturity) and the *w*/*c* ratio. The fastest dynamics (processes 1 and 2, representing very local water dynamics) are independent of water content and the degree of maturity whereas the slowest dynamics (processes 3 and 4) are dependent on the microstructure developed during curing. Additionally, we analyze the differences regarding the water dynamics when confined in synthetic C-S-H gel and in the C-S-H of Portland cement.

## Introduction

Portland cement (OPC) is one of the most common types of cement, and today is most commonly used construction material. In fact, every year more than 1 m^3^ is produced per person worldwide^1^. OPC consists of about two-thirds, by mass, of calcium silicates (3 CaO·SiO_2_ and 2 CaO·SiO_2_ (in cement notation C=CaO, S=SiO_2_; C3S and C2S respectively)) one-third of aluminum- (3 CaO · Al_2_O_3_ or C3A) and iron- (4 CaO · Al_2_O_3_ · Fe_2_O_3_ or C4AF) containing phases as well as other crystalline compounds. During the hydration process, water reacts with the cement particles (the so-called clinker) and several chemical and physical reactions take place. The developed structure is highly complex with different crystalline and semi-crystalline phases and other hydration products^2^. The main hydration product is a disordered semi-crystalline phase called calcium silicate hydrate (C-S-H), which bonds together all crystalline products. The water used for activation remains in the final product, either as an integral (chemically bound) part of the different components or confined in the porous structure. Water is thus a vital component of the cement structure^2, 3^. Moreover, important properties, such as shrinkage, creep, or durability^4^ of cements are attributed to the porous structure in which the water is confined.

Most of the structural models describing cements in fact describe the structure of C-S-H gel, which is the major component of these materials^5^. In principle, there are two types of approaches: colloidal- and layered-models. In the latter, water molecules are considered to be structurally incorporated in the C-S-H structure, which, in turn, is composed of an irregular array of single layers^6^. McDonald *et al*.^7^ proposed that water in a C-S-H gel pore can be immobile or mobile depending on the location of the water molecules (e.g. intra or inter the C-S-H sheets). On the other hand, the colloidal model proposed by Jennings^8, 9^ assumes that the microstructure of the cement paste is composed of a disk-like object (globule) having a layered internal structure similar to tobermorite and jennite. The packing of these globules produces a pattern in the gel in which pores of different sizes can be found (i.e. small 1–3 nm gel pores, and large 3–12 nm). In addition, cements also have capillary pores (also called macropores or mesopores) which are due to empty spaces after the hydration reaction takes place. Regardless of the model considered, the structure of hydrated cements is highly complex and comprises pores of different sizes in which water molecules can be located.

Molecular dynamics (MD) simulations have given important insights into describing the characteristics of water in cementitious materials. For instance, Manzano *et al*.^3^ performed MD simulations using the ReaxFF force field to show that a considerable amount of the water in C-S-H gel dissociates into H^+^ and OH^−^. In this scenario, the dissociated H^+^ and OH^−^ ions move to the nonbonding silicate oxygen atoms on the silicate chains (forming Si-OH groups) and to the inter-laminar Ca^2+^ ions (forming Ca-OH groups). This dissociation of water in C-S-H gel, of different Ca/Si ratios, was also studied by Hou *et al*.^10^. Recently Qomi *et al*.^11^ showed that the chemistry of the C-S-H affects the structural properties of water and that the motion of the water confined in the porous structure shows the same characteristics as supercooled and glassy liquids. The dynamical properties of water confined in cements, or in pores of pure C-S-H gel, have also been studied by different experimental techniques^12–17^. In particular, one of the most suitable techniques is broadband dielectric spectroscopy (BDS) because due to its large frequency range, it is possible to investigate water motion on different time scales and to obtain information about how the water molecules moves depending on the pore size^18–20^. This technique has previously been extensively used for analyzing the dynamics of water in the porous network of nano-alumina^21^, C-S-H gel^19, 20^, cements^18, 22^ as well as in other silica-based systems such as mineral clays^22, 23^, zeolites^24^, molecular sieves^25^ and MCM-41^26^.

The main aim of the present dielectric study is to analyze the dynamics of confined water in OPC at low temperatures, i.e. in a deeply supercooled regime. We analyzed samples of Portland cement prepared with different *w*/*c* ratios (0.3, 0.4 and 0.5) after 3 weeks and 4 years of preparation (OPC-3w and OPC-4y respectively). We found different relaxation processes, which can be attributed to different populations of water molecules located in the pores network developed during hydration. The more local processes do not change with *w*/*c* or aging time whereas the slowest processes show dependence on microstructure and water content. Additionally, we also compare and discuss differences and similarities with the dynamics of water confined in synthetic C-S-H-gel^19, 20^, MCM-41^26^ and in molecular sieves (10 Å)^25^. The latter is a material with similar structural characteristics to OPC.

## Results

### Characterization of materials

**Figure 1:**
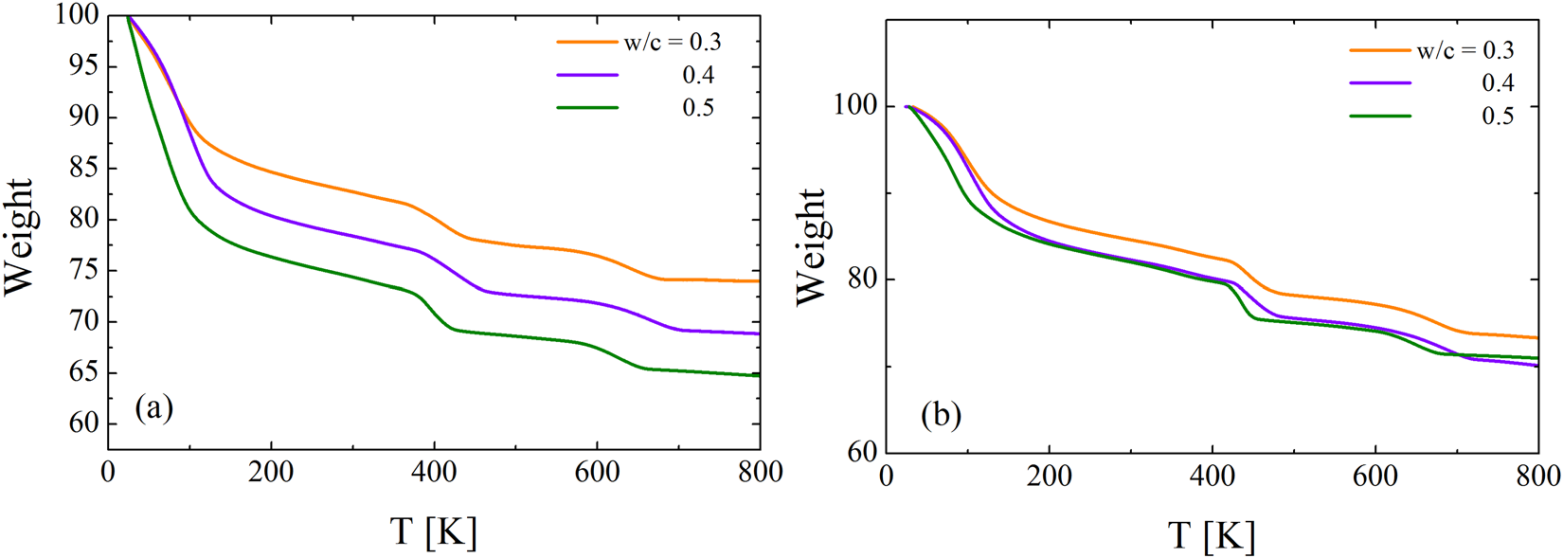
Thermogravimetric curves for different water-to-cement ratio (*w*/*c*) as indicated in the figure for samples after three weeks (**a**) and four years (**b**) of preparation.

Figure 1 shows the thermogravimetric response of Portland cement at different water-to-cement (*w*/*c*) ratios after three weeks (a) and four years (b) of preparation. Three different steps of weight loss as a function of temperature are found. Such behavior is typically observed in Portland cements and corresponds to the loss of water, dehydroxylation of portlandite^27^ and carbonates from low to high temperatures^28^. The water (c_w_) and the hydroxyl groups (c_OH_) contents of each sample were determined^29^ and the values are shown in Table 1. It should be noted that *c*_*w*_ was calculated as c_w_ = (w_40_-w_250_)/w_250_ (where w_40_ and w_250_ denote the weight of the sample at 40 and 250 °C respectively) and c_OH_ = (w_250_-w_500_)/w_500_ (where w_500_ denotes the weight of the sample at 500 °C). c_w_ represents the water (H_2_O) distributed throughout the sample (i.e. in capillary pores, and both small and big gel pores) whereas c_OH_ refers to the dehydroxylation of the rest of hydration products. Table 1 also shows *c*_*w*_ for the 4-years aged sample. *c*_*w*_ decreases after 4 years because both structural incorporation of water to the cement network and water evaporation from the capillary pores. In addition, the degree of hydration (*α*) for samples after 3 weeks and 4 years of preparation was estimated considering the work of Parrot *et al*.^30^ and the results are shown in Table 2.

**Table 1:** Water (H_2_O) (c_w_) and hydroxyl groups (c_OH_) contents obtained from TGA for the 3 weeks (OPC-3w) and aged for 4-years (OPC-4y). The degree of hydration (α) is also included.

*w*/*c*	3-weeks (OPC-3w)			4-years (OPC-4y)		
	c_w_ [wt %]	C_OH_ [wt %]	α	c_w_ [wt %]	c_OH_ [wt %]	α
0.50	30.10	9.80	0.77	20.50	10.50	0.81
0.40	24.50	9.20	0.87	20.50	10.10	0.88
0.30	18.10	8.00	0.88	17.00	9.30	0.88

**Table 2:** Activation Energy (*E*_*a*_) and Pre-Exponential Factor (log (*τ*_*o*_)) obtained from the Arrhenius equation applied to the data in Fig. 4.

Sample	*w*/*c*	Process 1		Process 2		Process 3 (145 K < T < 165 K)	
		log (τ_0_ [s])	*E*_*a*_ [*eV*]	log (τ_0_ [s])	*E*_*a*_ [*eV*]	log (τ_0_ [s])	*E*_*a*_ [*eV*]
OPC-3w	0.3	−13.5	0.19	−13.9	0.26	−15.0	0.42
	0.4	−13.3	0.20	−13.9	0.26	−16.4	0.47
	0.5	−13.9	0.20	−13.8	0.26	—	—
OPC-4y	0.3	−14.5	0.23	−14.6	0.28	−13.3	0.39
	0.4	−13.6	0.21	−14.5	0.28	−12.9	0.37
	0.5	−12.5	0.18	−14.4	0.28	−14.1	0.41

### Dielectric response of Portland cement – Fitting procedure

In a dielectric experiment, the interaction between an external electric field and the permanent molecular dipoles within the material under investigation is measured^31^. Through measurements of complex permittivity (ε*(*ω*) = *ε*′(*ω*) − *ιε*″(*ω*)) it is possible to analyze the dynamics of dipolar species as well as the charge transport. In the case of Portland cement, water molecules and hydroxyl groups are expected to contribute to the dielectric signal in a similar way as previously observed for synthetic C-S-H gel (developed by hydration of single C_3_S and hereafter referred to as C-S-H_C3S_)^19, 20^.

**Figure 2:**
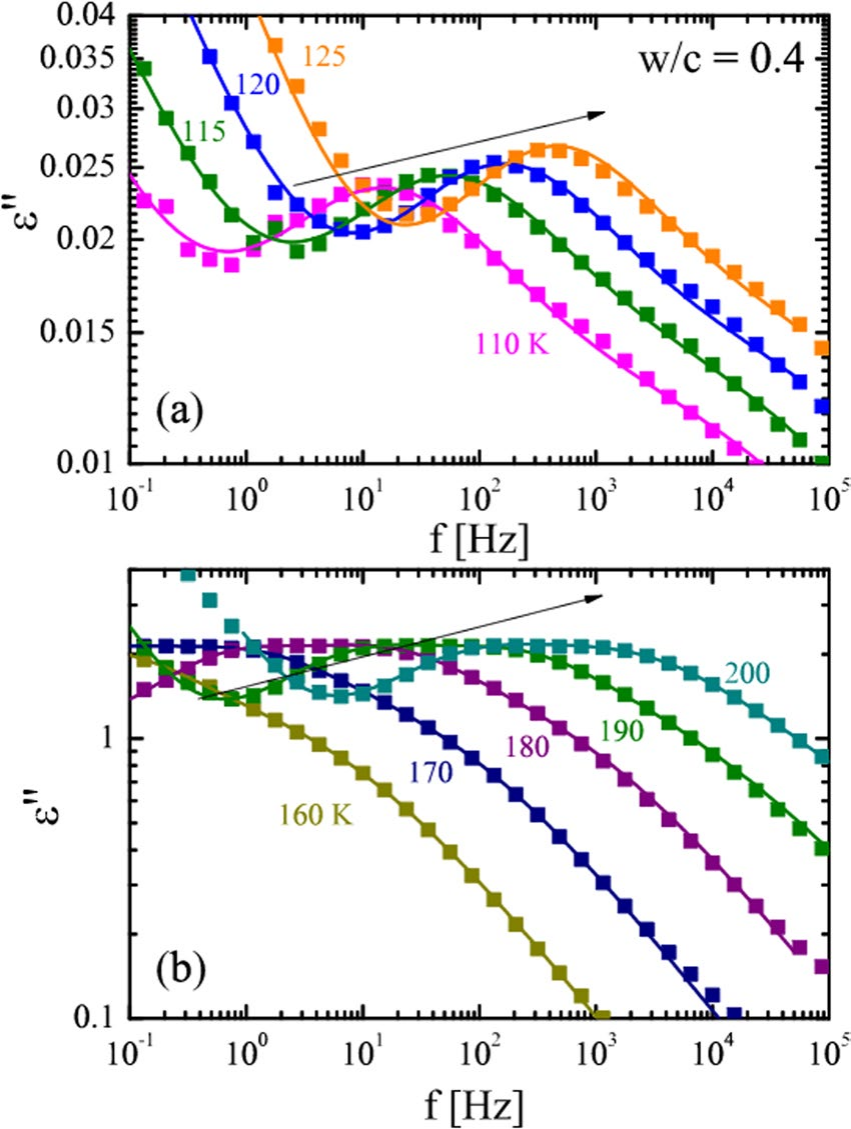
(**a** and **b**). Imaginary part (ε″) of the dielectric spectra of Portland cement after three weeks of preparation with *w*/*c* = 0.4 at different temperatures (see plot). The solid lines through the data points represent the fits to the experimental data.

All the samples presented two broad temperature-dependent dielectric processes (see arrows in Fig. 2). The different dielectric processes shift to higher frequencies with increasing temperature. Starting from the low temperature region (110 to 150 K), the asymmetry of this component suggests the presence of two different dynamical processes. Increasing the temperature (160 to 250 K), a stronger process enters into the frequency window. Due to the broad and asymmetric shape of the peaks in Fig. 2, the data suggest that such components are also originated by more than a single process.

The dielectric response can be described by using standard fit functions. As commonly done for water in confinements, the symmetric Cole–Cole (CC) function^32^ is used to describe each relaxation process,

1 $${\varepsilon }^{\ast }(\omega )={\varepsilon }_{\infty }+\frac{{\rm{\Delta }}\varepsilon }{1+{(i\omega \tau )}^{\alpha }}$$

where *Δε* is the dielectric strength (*Δε* = *ε*_*s*_ *−* *ε*_*∞*_, *ε*_*∞*_ and *ε*_*s*_ are the unrelaxed and relaxed values of the dielectric constant respectively), *τ* is the relaxation time, *α* is the stretching parameter and *ω* = 2*πf* is the angular frequency. In addition, at low frequencies and high temperatures, the spectra are dominated by conductivity effects and therefore a power law term was added (*σ*/(*ε*_0_ *ω*)) where ε_0_ denotes the vacuum permittivity and σ is the static ionic conductivity.

**Figure 3:**
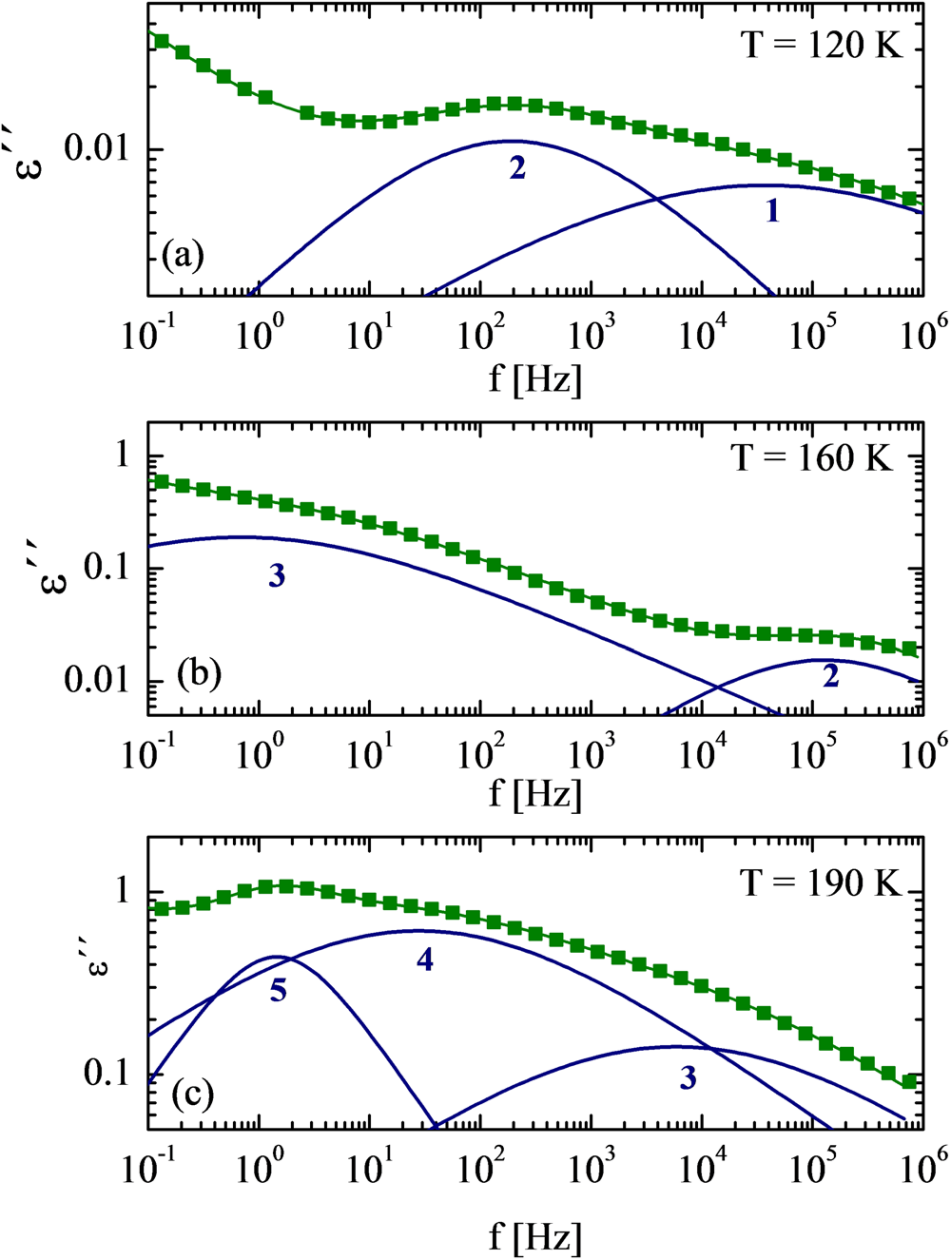
Imaginary part (*ε*″) of the dielectric spectra of Portland cement at *w*/*c* = 0.3 after three weeks of preparation. (**a**) T = 120 K and (**b**) T = 160 K, (**c**) T = 190 K. The solid lines through the data points represent the fits to the experimental data. Individual processes (1 to 5) for sample *w*/*c* = 0.3 are also shown.

For all the samples analyzed (OPC-3w and OPC-4y), in the low temperature range (100 to 150 K), two Cole-Cole functions (processes 1 and 2) were used to fit the data, whereas at higher temperatures (150 to 250 K) the combination of three C-C functions was needed to describe the data (processes 3, 4 and 5). The exception is the sample OPC-3w (*w*/*c* = 0.5) in which conductivity effects prevent the clear observation of these three dielectric processes. Figure 3 shows the dielectric loss (*ε*″) and the fitting curves at three different temperatures (110 K (a), 160 K and 190 K (b)) for OPC-3w at *w*/*c* = 0.3. In the following, we will compare the main fitting parameters (relaxation times and relaxation strength) for the different *w*/*c* ratios and aging times.

### Temperature dependence of the relaxation times of OPC

**Figure 4:**
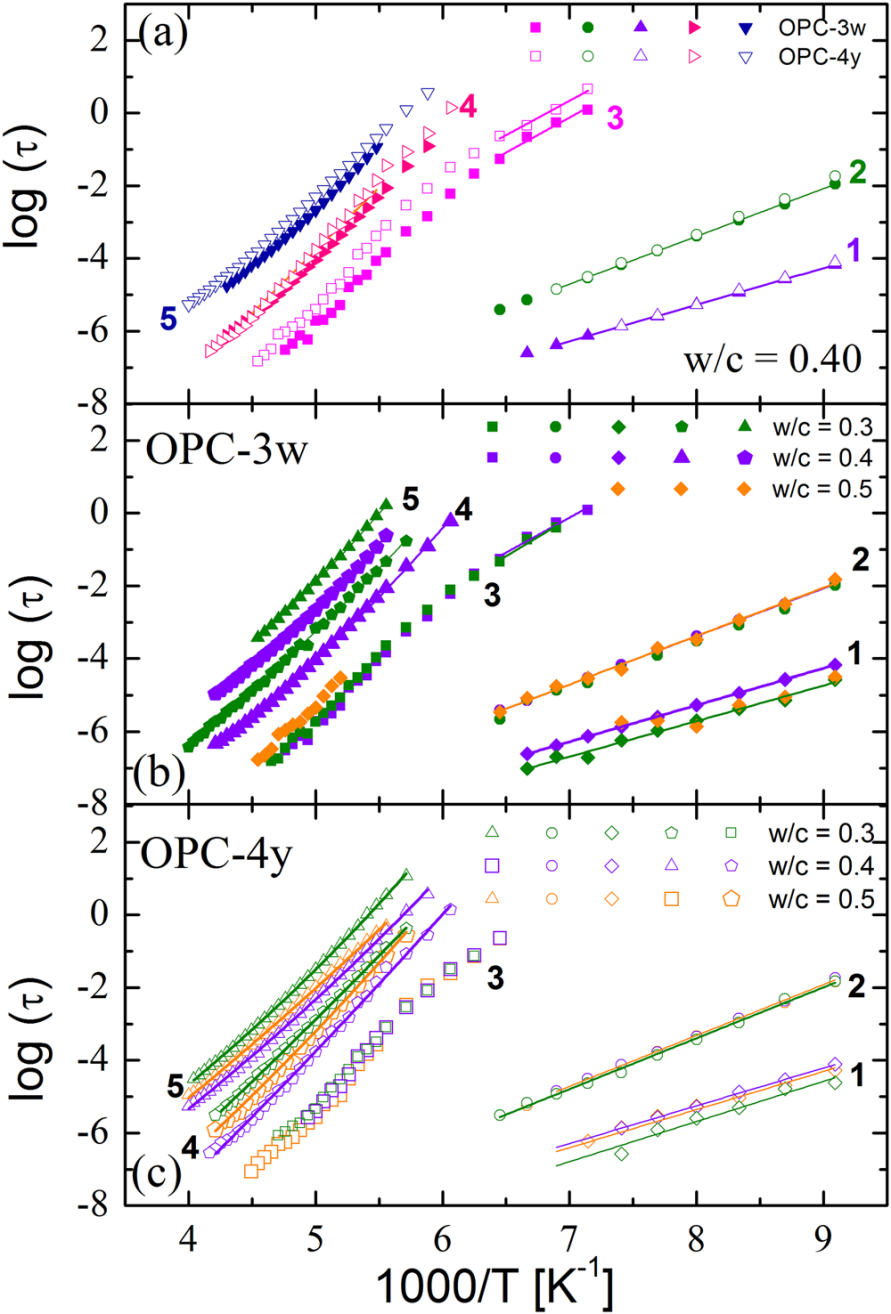
(**a**) Temperature dependence of relaxation times of water confined in OPC *w*/*c* = 0.4 after 3 weeks and 4 years of preparation. (**b** and **c**) Temperature dependence of relaxation times of water confined in cements with different *w*/*c* ratios after 3 weeks (**b**) and 4 years (**c**) of preparation.

Figure 4a shows the temperature dependence of the relaxation times of the five dynamics of OPC-3w and OPC-4y at *w*/*c* = 0.4. In addition in Fig. 4b and c, we compare the temperature dependence of the relaxation times for different *w*/*c* ratios of OPC-3w and OPC-4y respectively.

Processes 1 and 2 show an Arrhenius-type temperature dependence in the entire temperature range (τ = τ_0_ *exp*(*E*_*a*_/*kT*), where *E*_*a*_ represents the activation energy for the reorientation of the relaxing dipole, and log (*τ*_0_) is a pre-exponential factor, (see values of *E*_*a*_ and log(*τ*_0_) in Table 2)). Relaxation times of processes 1 and 2 are independent of both the aging time (Fig. 4a) and the *w*/*c* ratio (Fig. 4b and c). This indicates that water relaxing via processes 1 and 2 are independent from the pore network developed during hydration.

Process 3 exhibits two different types of behavior depending on the temperature. Below 165 K, the process 3 follows Arrhenius behavior (see values of E_a_ and log (τ_0_) in Table 2) and, above this temperature, there is a systematic reduction of the relaxation times with respect to the line extrapolated from the low temperature behavior. This change in the temperature dependence of the relaxation time has previously been observed for water confined in different confining systems and extensively debated in a previous publication^33^. In the case of synthetic C-S-H gel^34^, this crossover was related to confinement sizes of 1 nm. Moreover, the relaxation time of process 3 is independent of the *w*/*c* ratio (i.e. water content). The independence of relaxation times on water content is typical of the relaxation of water under confinement in small pores^35^ and therefore a strong indication that this process reflect the relaxation of confined water. Therefore, process 3 represents the dynamics of water confined in the small gel pores (∼1 nm) of Portland cement. These pores are located in the C-S-H gel phase of OPC and denoted here as C-S-H_OPC_.

The relaxation time of process 3 becomes slightly slower for the aged samples (see Fig. 4a). It is important to note that the OPC here analyzed was maintained in the same preparation container for 4 years at room temperature. The variation of the relaxation time of process 3 with aging time is therefore related to the changes in pores sizes as hydration proceeds.

Contrary to the fastest processes 1–3, the relaxation time of processes 4 and 5 depends on the *w*/*c* ratio (see Fig. 4b and c) and the aging time (see Fig. 4a). Therefore these relaxation processes are related with the structural changes produced during cement hydration. The temperature dependence of processes 4 and 5 follow a Vogel-Fulcher-Tamman (VFT) temperature dependence^36–38^ (τ (T) = τ_0_ exp (D T_o_/(T − T_0_)), where τ_0_, *D* and *T*_0_ are fitting parameters). All these parameters are shown in Table 3 for all the samples analyzed.

**Table 3:** VFT parameters for processes 4 and 5 showed in Fig. 4.

Sample	w/c	Process 4			Process 5		
		D	To [K]	log (τ_o_ [s])	D	To [K]	log (τ_o_ [s])
OPC-3w	0.3	31.5	83.1	−13.1	27.2	90.2	−11.6
OPC-3w	0.4	21.0	94.5	−12.2	24.3	92.0	−11.6
OPC-4y	0.3	37.7	78.5	−13.5	59.5	63.0	−13.4
OPC-4y	0.4	33.0	84.7	−13.8	44.5	72.0	−12.4
OPC-4y	0.5	28.9	89.0	−13.9	31.6	81.9	−11.9

### Temperature dependence of the relaxation strength of OPC

**Figure 5:**
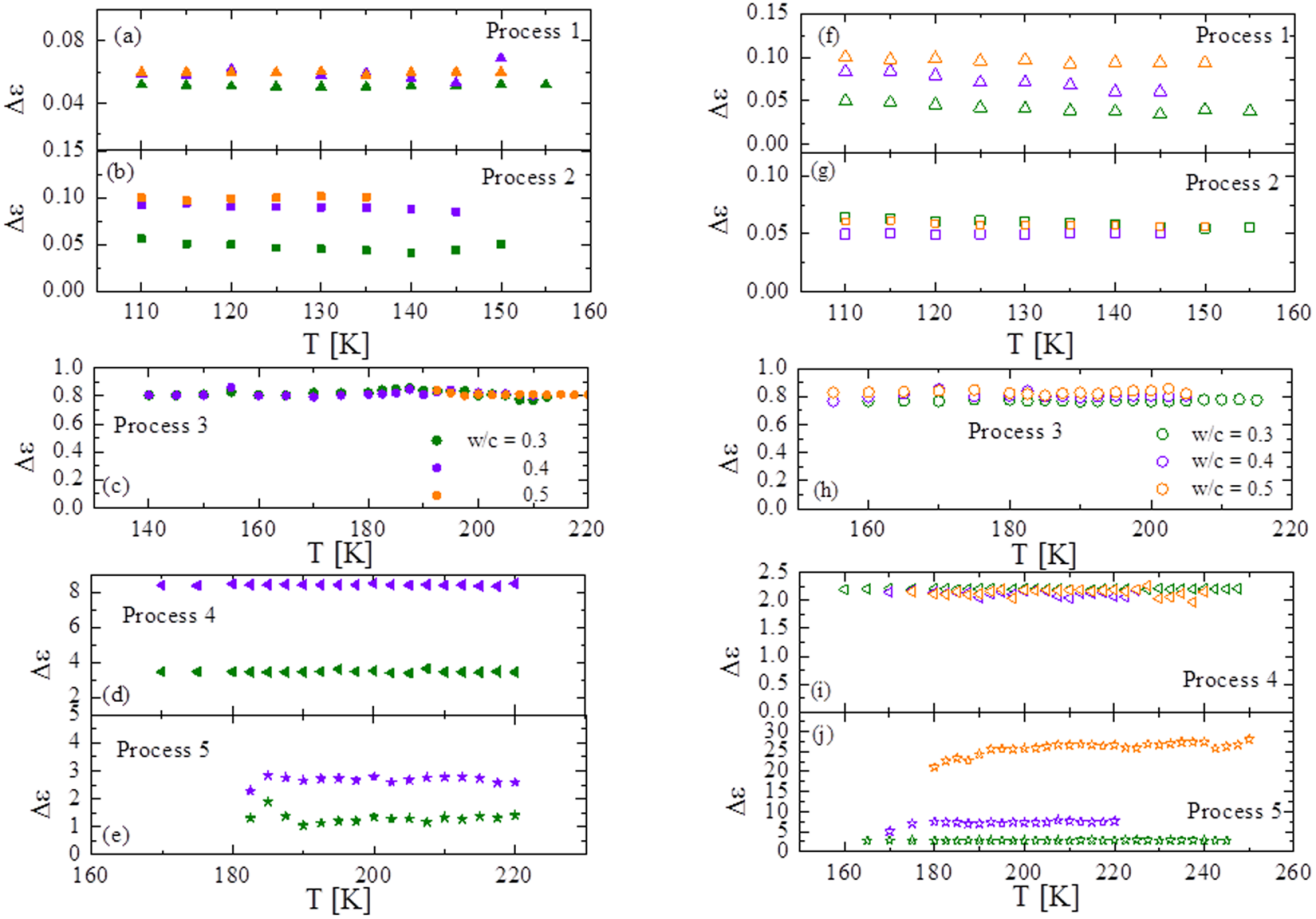
Temperature dependence of relaxation strength (*Δε*) of processes 1 to 5 for samples OPC-3w (left panel) and OPC-4y (right panel).

Figure 5 shows the relaxation strength, *Δε*, as a function of the temperature for the different *w*/*c* ratios of OPC-3w and OPC-4y. *Δε* is related to the number of water dipoles participating in relaxation as well as its ability for reorientation. *Δε* of processes 1 to 3 are independent of the total water content determined by TGA, the *w*/*c* ratio and the aging time. This indicates that the number of water molecules relaxing via these three processes is essentially the same, independent of the details of the pore network and even of the structural changes produced during aging.

Regarding the relaxations at higher temperatures, the dielectric strength of process 4 depends on both the *w*/*c* ratio and the aging time. The water molecules that originate this process are therefore located in pores that undergo some evolution during hydration (either the pore size or the water content). Finally, the relaxation strength of process 5 increases with decreasing water content. This behavior indicates that this contribution is the so-called Maxwell-Wagner-Sillars (MWS) polarization^31^. Recently Abe *et al*.^18^ have analyzed mortar samples at higher temperatures than those here analyzed. They observed a dielectric process (called in their publication “process 3”) with similar characteristics as those observed here in process 5. In fact, drying the mortar sample this process gradually shifts to lower frequencies and finally disappear from the frequency window. This behavior is normally observed in the MWS polarization^39^. For this reason, process 5 is not further discussed in the present work.

### Dielectric response of water under different confinements vs water confined in OPC

We now compare the relaxation times of water confined in C-S-H_OPC_ and C-S-H_C3S_ with that obtained in other silicate systems such as molecular sieves^25^ and MCM-41^26^. Both MCM-41 and molecular sieves (MS) are made up aluminosilicates, with a three-dimensional interconnecting structure of silica and aluminum tetrahedrals^40, 41^. This is a similar structure to that of hydrated cement. MCM-41 has cylindrically shaped pores which do not greatly perturb the wall structure, the shapes, and the topology of the porous network. The structure of MCM-41 has a rather well-defined pore size distribution. By contrast, the structure of molecular sieves is more disordered with a network of short worm-like channels. Molecular sieves are therefore more similar to the irregular and disordered structure of cements^42^. Therefore, it is not surprising that the dielectric responses of the different types of C-S-H´s gels (C-S-H_OPC_ and C-S-H_C3S_) show behavior corresponding to these two model systems although the topology of the porous network of cement materials is far more complex. In hydrated cements, there are different multiple crystalline hydrates phases (for instance portlandite or ettringite) immersed in the C-S-H gel matrix, which in turn also has a complex pore distribution^43^.

**Figure 6:**
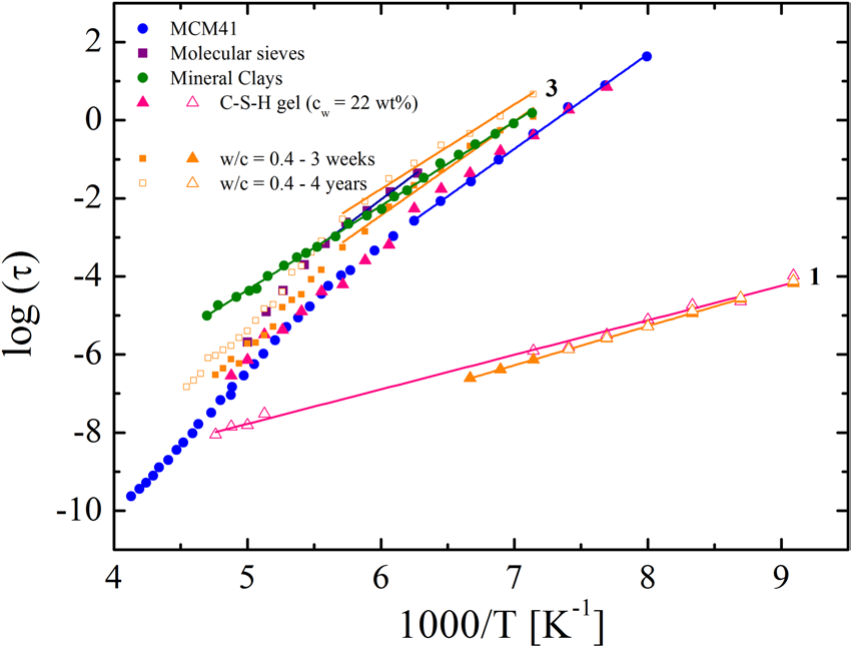
Comparison of the temperature dependence of relaxation times of water confined in Portland cements with different *w/c* ratio, in C-S- H gel^20^, molecular sieves (1 nm)^51^, MCM-41 (1 nm)^26^ and mineral clays^52^.

Figure 6 shows a comparison between the relaxation times of process 3 in C-S-H_OPC_ and those observed in C-S-H_C3S_ at similar water content, together with water confined in molecular sieves^25^ and MCM-41^26^. Two different scenarios can be observed, one for C-S-H_C3S_ and MCM-41 and other for C-S-H_OPC_ and molecular sieves.

The relaxation time of water confined in the molecular sieves reflects the dynamics in an average pore size of 1 nm^25^, and therefore process 3 in C-S-H_OPC_ is related to water in disordered pores of about the same size. This assumption is further supported by the fact that the temperature dependence of the relaxation time of process 3 exhibits a crossover around ~175 K and the activation energy is similar to that found in molecular sieves. Such a crossover is associated with confinement effects^44^, produced by the small size of the pores in which the water is situated^45^. On the other hand, the temperature dependence of the relaxation times of MCM-41 is similar to that of C-S-H_C3S_.This reflects a more ordered pore network developed in C-S-H_C3S_ than in OPC.

## Discussion

As mentioned in the previous section, the temperature dependence of the relaxation times of processes 1 to 3 in C-S-H_OPC_ does not vary with the *w*/*c* ratio (i.e. water content) as normally observed for water in other confinement systems^45^. This behavior contrasts with water confined in C-S-H_C3S_ (i.e. synthetic C-S-H developed by hydration of single C_3_S), where a significant variation of the relaxation times with water content was observed^19^. This gives a strong indication of the large structural differences between C-S-H_C3S_ and C-S-H_OPC._ This is further supported by the fact that process 3 is faster in C-S-H_C3S_^19^ than in C-S-H_OPC_ with the same water content (see Fig. 6). The structure of hydrated Portland cement contains disordered C-S-H gel, and also other hydration products and crystalline phases. In addition, the Ca/Si ratio of the C-S-H_OPC_ (Ca/Si = 2.5) investigated here is much higher compared to the previously investigated C-S-H_C3S_ (Ca/Si = 1.3), which is a factor that is known to have a great influence on the structural properties of cements. Whereas the structure of a material with a high Ca/Si ratio is disordered, an ordered structure is observed in materials with a low Ca/Si ratio^46^. With increasing the Ca/Si ratio, the number of bridging groups decreases, and consequently, the length of the silicate chains becomes shorter. Therefore, for higher Ca/Si ratios a larger amount of dangling oxygen (Si-O^−^) is present in the structure than for lower Ca/Si ratios^46^. This indicates that shorter chains should result in a reduced variation in pore sizes since smaller pores and cavities in the sheets to a large extent become more connected^11^. Thus, in the case of C-S-H_C3S_ a more ordered and well-defined structure than for Portland cement is expected.

The relaxation strength of process 3 has no dependence with aging or water content. Therefore, it is possible to conclude that the smallest pores are saturated by water from 3 weeks to 4 years (the number of water molecules located in these small pores is not appreciable changing with aging).

The relaxation strength of process 4 depends on the *w*/*c* ratio and therefore on the microstructure development of cement. In addition, this process is almost two orders of magnitude slower than process 3. This behavior can be explained if we assume that water molecules giving rise to this process 4 are located in pores larger than 1 nm (which is the origin of process 3) as also suggested in our previous work of C-S-H_C3S_^19^. In accordance with this interpretation, the temperature dependence of the relaxation time does not follow Arrhenius behavior indicating that the number of water molecules relaxing via process 4 is big enough to show more cooperative behavior i.e. pores bigger than 1 nm. In agreement with this analysis, Abe *et al*.^18^ also observed a relaxation process (called “process 2” in their work) compatible with our process 4.

The activation energy of processes 1 and 2 are ~0.20 and ~0.28 eV, respectively, and they are independent of the *w*/*c* ratio. These values are much lower than the activation energy of a more collective behavior of confined water (which is normally around 0.5 eV)^44^. This indicates that, in the present case, a less extensive and weaker hydrogen bonded network is formed. Following that both the relaxation time and the relaxation strength of processes 1 to 2 are independent of water content and the aging time, these processes reflect very local water dynamics.

The relaxation times for process 1 are very similar in both C-S-H_C3S_ and C-S-H_OPC_ (Fig. 6), which suggests the same origin in both cases. Since process 1 in C-S-H_C3S_ was due to the rotation of hydrated hydroxyl groups (OH) on the internal surface of the material^20, 39^, we also assign the same origin to process 1 in hydrated C-S-H_OPC_.

The relaxation times of process 2 are significantly different to those of processes 1 and 3, which indicates that a different water population is the origin of this process. Despite the fact that C-S-H_C3S_ has a larger variation of the pore sizes than C-S-H_OPC_, only the two processes (corresponding to process 1 and 3) were observed for water in C-S-H_C3S_. Moreover, process 2 has low activation energy (0.26 eV) and it does not show the typical crossover in temperature dependence usually observed for water in pores^47^. The internal environment of cements is alkaline with a relatively high pH. As for other silica based porous materials^48^, the pH has an influence on the internal structure of cements. At high pH there is a reduction of surface hydroxyl groups and an increase of dangling oxygen groups (Si-O^−^), with an increased surface charge as a consequence. In addition, the substitution of aluminum (Al^3+^) in the silicate structures (Si^4+^) also affects the surface charge which is compensated by the presence of calcium ions (Ca^2+^) located close to the surface. Thus, the water dynamics in cements is not only affected by the confining geometry in pores but also by the large amounts of calcium cations (Ca^2+^) in the structure. Ca^2+^ attracts water^49^ and therefore it has a large structural effect on water molecules^48, 49^. In addition, their presence increases the hydrophilicity of the porous environment. This in turn, has a huge reducing influence on the time scale of water relaxation^48^. Thus, with increased hydrophilicity in the porous structure, water molecules reside for longer times compared to water in regions with lower hydrophilicity. In addition, the more structured water surrounding the calcium ions relaxes faster than free water molecules. Since the time scale in process 2 is much faster than process 3 and also much slower compared to process 1 (OH-group reorientation), we attribute this process to be due to water surrounding the charge compensating calcium cations close to the surface of the internal structures. The reason why this process was not observed in previous investigations on C-S-H_C3S_ is most likely that material contains a lower content of Ca^2+^ due to the lower Ca/Si ratio (with a lower fraction of Si-O^−^ and lower pH value as consequences). Finally, the activation energy obtained for water confined in montmorillonite^24, 50^ with two types of cations (Ca^2+^ and K^+^) is similar to that observed here for process 2 although the time scale of both processes are different. In that work, the authors also assumed that this relaxation process reflects the water structure near the hydrated cations located in the interlayer in agreement with our interpretation for the process 2.

### Summary of dielectric processes measured in Portland cement

Portland cement is an extremely heterogeneous material and therefore several processes can be detected using dielectric spectroscopy. Here we summarize the origin and characteristics of each process:

1. Process 1 is originated in the rotation of hydrated hydroxyl groups (OH) on the internal surface of the Portland cement.
2. Process 2 is related to the relaxation of water molecules surrounding the charge compensating calcium cations.
3. Process 3 is due to relaxation of water molecules in the small gel pores of C-S-H_OPC_ (1–3 nm).
4. Process 4 is due to relaxation of water molecules in the big gel pores of C-S-H_OPC_.
5. Process 5 is the Maxwell-Wagner-Sillars polarization.

Processes 1 and 2 reflect a very local dynamics (i.e. they relaxation times have Arrhenius temperature dependence) and both processes are independent on the w/c ratio or aging time and therefore independent on the pore network developed during hydration. On the other hand, processes 3 and 4 reflect the dynamics of water in bigger pores and therefore more cooperativity of water molecules can be detected by dielectric spectroscopy (i.e. VFT behavior). These processes are dependent on the *w*/*c* ratio and aging time and therefore they are dependent on the microstructure of cements.

Other important finding is the fact that water confined in C-S-H_OPC_ and C-S-H_C3S_ has different dynamical behavior and this reveals the large structural differences between these two materials.

## Methods

### Experimental Techniques

Thermogravimetric analysis was performed using a TA Instruments Q500 at a heating rate of 5 K/min^−1^ under nitrogen flow to determine the contents of water and portlandite and the degree of carbonation.

A broadband dielectric spectrometer, Novocontrol Alpha-N, was used to measure complex dielectric permittivity, ε* (*ω*) = *ε*′ (*ω*) − *i ε*″ (*ω*) (*ω* = 2*π* *f*), in the frequency range 10^−1^–10^6^ Hz. The isothermal frequency scans were performed on heating every 5 degrees over the 110–250 K temperature range. At higher temperatures than 250 K, the relaxation processes approach the limit of the experimental window and therefore all the relaxation processes are out of the experimental window. The sample temperature was controlled with stability better than ±0.1 K. Parallel gold plated electrodes (with a diameter of 30 mm and a sample thickness of 0.4 mm) were used.

### Samples

The material used in this study was Portland cement (Cementa, Heidelberg Cement Northern Europe Sweden). The mineral composition determined by XRD-Rietveld analysis is given in Table 4. Samples at different water-to-cement ratios by mass (*w*/*c* = 0.3, 0.4 and 0.5) were prepared using distilled water. To avoid water evaporation and sedimentation during hardening, each sample was casted in a sealed plastic tube (diameter 2 cm), which was slowly rotated during 2 days. After 1 week of hydration, circular samples were cut from the hardened material and then stored in plastic bags at room temperature (298 K). The analysis of the samples was done 3 weeks after the preparation casting (OPC-3w) and after four years (OPC-4y). It is important to note that the OPC was maintained in the same preparation container for 4 years at room temperature.

**Table 4:** Mineral composition determined by XRD-Rietveld analysis of the cement studied in this work.

Mineral	[wt %]
C3S	74.8
C2S	5.8
C4AF	4.4
C3A	0.8
Calcite	9.0

Hydration products in the sample aged during 4-years were determined using XRD-Rietveld analysis and the following phases were found: calcite (55.4 wt%), Portlandite (36.6 wt%), AFt (6.6 wt%) whereas AFm was not quantifiable. The oxide contents of the materials (Table 5) were determined by X-ray fluorescence. From these results and TGA measurements the Ca/Si ratio was determined to 2.5 for all samples.

**Table 5:** Chemical composition [wt%] obtained through X-ray fluorescence analysis for Portland cement (OPC).

	SiO_2_	Al_2_O_3_	Fe_2_O_3_	MnO	MgO	CaO	Na_2_O	K_2_O	TiO_2_	P_2_O5	SO_3_
OPC	14.54	2.93	2.20	0.03	1.83	48.20	0.07	0.72	0.22	0.04	1.78

The datasets generated during and/or analyzed during the current study are available from the corresponding author on reasonable request.
